# The Effect of Phosphodiesterase-type 5 Inhibitors on Erectile Function: An Overview of Systematic Reviews

**DOI:** 10.3389/fphar.2021.735708

**Published:** 2021-09-07

**Authors:** Nikolaos Pyrgidis, Ioannis Mykoniatis, Anna-Bettina Haidich, Maria Tirta, Persefoni Talimtzi, Dimitrios Kalyvianakis, Andreas Ouranidis, Dimitrios Hatzichristou

**Affiliations:** ^1^ Institute for the Study of Urological Diseases, Thessaloniki, Greece; ^2^ Urology Department, School of Medicine, Faculty of Health Sciences, Aristotle University of Thessaloniki, Thessaloniki, Greece; ^3^ Department of Hygiene, Social-Preventive Medicine and Medical Statistics, Medical School, Aristotle University of Thessaloniki, Thessaloniki, Greece; ^4^ Department of Pharmaceutical Technology, Aristotle University of Thessaloniki, Thessaloniki, Greece; ^5^ Department of Chemical Engineering, Aristotle University of Thessaloniki, Thessaloniki, Greece

**Keywords:** phosphodiesterase type 5 (PDE5) inhibitors, overview of systematic reviews, erectile dysfucntion, meta-analysis, sexual dysfuction

## Abstract

**Background:** Multiple systematic reviews explore the effect of phosphodiesterase type 5 (PDE5) inhibitors on erectile dysfunction (ED), with each study addressing specific outcomes. However, physicians and policymakers require a holistic approach of this topic.

**Objective:** To summarize the current evidence regarding the efficacy and safety of PDE5 inhibitors for the management of ED through an overview of systematic reviews.

**Methods:** Studies were identified by searching PubMed, Web of Science, Cochrane Library and Scopus databases, as well as sources of grey literature until June 12, 2021 (PROSPERO: CRD42020216754). We considered systematic reviews, meta-analyses or network meta-analyses of randomized trials that provided outcomes about the efficacy and safety of any approved PDE5 inhibitor (avanafil, sildenafil, tadalafil and vardenafil). We constructed forest plots for meta-analytic effects regarding the change in erectile function, adverse events and dropouts after administration of PDE5 inhibitors in the general population and in specific patient groups.

**Results:** We included 23 studies with 154,796 participants and a total of 258 meta-analytic effects. Sildenafil 25 mg [Weighted Mean Difference (WMD): 13.08, 95% Confidence Interval (CI): 10.1-16.06] seemed to be statistically superior to all interventions in improving erectile function compared to placebo, but studies with low-dose sildenafil are lacking. Moreover, comparing among different PDE5 inhibitors, sildenafil 50 mg or sildenafil 100 mg were considered the most effective compounds in the general population. The latter derived, however, predominantly from indirect comparisons among different PDE5 inhibitors. Still, sildenafil 100 mg was associated with more treatment-related adverse events and dropouts. Interestingly, low-dose daily tadalafil may be more effective than high-dose on-demand tadalafil (WMD: 1.24, 95% CI: 0.03-2.44). Furthermore, testosterone and PDE5 inhibitors in patients with ED and hypogonadism seem to further improve symptoms, while the addition of a-blockers in patients with urinary symptoms treated with PDE5 inhibitors does not provide additional benefits (WMD: −0.8, 95% CI: −1.65-0.06).

**Conclusion:** Although the efficacy and safety of PDE5 inhibitors, compared to placebo, is well-documented, the existing evidence comparing different PDE5 inhibitors is low. Therefore, high-quality, head-to-head, trials comparing different PDE5 inhibitors are necessary to determine their ideal dosage and formulation based on their safety and efficacy profile.

**Systematic Review Registration**: PROSPERO, identifier [CRD42020216754].

## Introduction

Phosphodiesterase type 5 (PDE5) inhibitors are considered the first-line treatment in patients with erectile dysfunction (ED) due to their safety and efficacy profile (Salonia et al., 2021). To date, seven PDE5 inhibitors exist (avanafil, lodenafil, mirodenafil, sildenafil, tadalafil, udenafil, and vardenafil) and four of them (avanafil, sildenafil, tadalafil, and vardenafil) are currently approved by the EMA and FDA for the management of ED (Goldstein et al., 2019). Established evidence indicates that PDE5 inhibitors are also effective and safe across multiple subgroups of patients with difficult-to-treat ED (Burnett et al., 2018). Similarly, PDE5 inhibitors seem to display beneficial outcomes when they are combined with other effective treatment modalities (Dhir et al., 2011).

Due to the increasing interest in PDE5 inhibitors, available systematic reviews and meta-analyses have explored the effect of PDE5 inhibitors on multiple outcomes (Greenberg et al., 2019). Hence, each systematic review and meta-analysis addresses a specific outcome. On the contrary, physicians and policymakers require, in most cases, a holistic approach of a given topic to facilitate their evidence-based clinical decision-making (Allen and Walter, 2019). In this scope, overviews of systematic reviews and meta-analyses or umbrella reviews are a promising, new approach that assimilates the vast amount of available research and contextualizes the magnitude of a specific topic (Hunt et al., 2018). These studies are growing in popularity, as they provide high level of recommendations and showcase potential gaps in the literature, by compiling the results of different systematic reviews, meta-analyses and network meta-analyses (McKenzie and Brennan, 2017).

Within this framework, we performed an overview of systematic reviews aiming to summarize the current evidence regarding the efficacy and safety of PDE5 inhibitors for the management of ED.

## Methods and Analysis

### Data Sources and Searches

All findings in our study are reported in accordance with the PRIO-harms guidelines (Bougioukas et al., 2018; Bougioukas et al., 2019). We published our predefined protocol after registering the aims and methods of this overview of systematic reviews at PROSPERO (CRD42020216754) (Pyrgidis et al., 2021).

Two authors (NP, MT) systematically searched PubMed, Web of Science, The Cochrane Library and Scopus databases from inception to June 12, 2021. They also perused the reference lists of all identified studies, as well as potential sources of grey literature, including conference abstracts published in relevant journals and websites for healthcare evidence such as epistemonikos.org. The detailed search strategy is depicted in Supplementary Data S1.

### Selection Criteria

We included systematic reviews with or without meta-analysis in adults with ED performing heterosexual activity that: i) compared the efficacy and safety of any dose of PDE5 inhibitors with another PDE5 inhibitor, with placebo or with other effective treatments; ii) provided outcomes of interest deriving from randomized controlled trials (RCTs); iii) explored the use of any approved PDE5 inhibitor by the EMA and FDA (avanafil, sildenafil, tadalafil, and vardenafil) alone or in combination with other treatment modalities both in the general male population, as well as in specific patient groups and; iv) were conducted based on the Cochrane Handbook for Systematic Reviews of Interventions and the PRISMA statement. On the other hand, we excluded: i) systematic reviews or meta-analyses assessing the efficacy and safety of PDE5 inhibitors for indications not relevant to erectile function and; ii) narrative reviews, editorials or letters to the editor.

When we identified both systematic reviews and meta-analyses addressing similar outcomes, the meta-analyses were only included, given that they included more primary studies. Similarly, when we identified systematic reviews or meta-analyses and network meta-analyses addressing similar outcomes, the network meta-analyses were only included, given that they included more primary studies. When we identified studies with the same design (systematic reviews or meta-analyses or network meta-analyses) addressing similar outcomes, ideally the most recent study or, otherwise, the most methodologically rigorous study, based on quality assessment, was included (Cooper and Koenka, 2012). Therefore, for each outcome, we included only one study, preferably a network meta-analysis followed by a meta-analysis and a systematic review.

### Data Extraction and Quality Assessment

Two authors (NP, MT) independently performed a three-step parallel review of title, abstract and full text of all retrieved records based on our predetermined selection criteria. All records excluded at the level of full text evaluation were saved and presented in Supplementary Data S2. Any discrepancies throughout the screening process were resolved by consensus. Data extraction was undertaken independently in a Microsoft Excel spreadsheet. The two authors tabulated information regarding study characteristics, intervention details and outcomes. They also performed a pilot test before data extraction to ensure the coherence of the procedure (Higgins et al., 2019). In studies evaluating the effect of PDE5 inhibitors on erectile function with multiple questionnaires, data regarding the International Index of Erectile Function (IIEF)–Erectile Function Domain or IIEF-5 were only extracted.

We employed the AMSTAR 2 tool to assess the quality of all included systematic reviews, meta-analyses or network meta-analyses (Shea et al., 2017). The extent of overlapping among included studies was estimated based on the corrected covered area (CCA) and was presented with novel graphical approaches (Pieper et al., 2014; Bougioukas et al., 2021). The strength of evidence for each meta-analytic effect was determined based on the Grading of Recommendations Assessment, Development and Evaluation (GRADE) (Guyatt et al., 2008). In particular, we evaluated the strength of evidence of each meta-analytic effect based on the corresponding results from the systematic review.

### Outcomes and Data Analysis

The primary outcome of our overview was the mean change in the erectile function after PDE5 inhibitor intake in the general population measured with the IIEF-5 or the IIEF–Erectile Function Domain. Secondary outcomes included: i) mean change in the erectile function after PDE5 inhibitor intake in specific patient groups based on data availability measured with the IIEF and; ii) severe adverse events and dropouts after PDE5 inhibitor intake both in the general population, as well as in specific patient groups based on data availability.

We performed a descriptive analysis of all included studies. For studies performing meta-analyses or network meta-analyses, we constructed forest plots with the corresponding confidence interval (CI) for all relevant meta-analytic effects. In particular, continuous effect estimates were presented in the form of standardized mean difference (SMD) or weighted mean difference (WMD), while categorical effect estimates were presented in the form of odds ratio (OR). When we identified studies that performed meta-analyses with a fixed effects model, we reanalyzed all outcomes using the DerSimonian and Laird random effects model.

For each outcome, heterogeneity was evaluated with the I^2^ and publication bias was estimated with the Egger’s statistical test. Due to the plethora of primary studies included in each meta-analysis, all relevant measures were presented as they were reported in each study without reviewing the corresponding primary studies (Aromataris et al., 2015). All analyses were undertaken using Microsoft Excel (Version 16.42) and R statistical software (version 3.6.3).

## Results

### Study Selection and Characteristics

Our literature search yielded 686 potentially eligible unique systematic reviews. Ultimately, we included 23 systematic reviews with 563 primary studies and 154,796 participants (Shabsigh et al., 2007; Vecchio et al., 2010; Xiao et al., 2012; Taylor et al., 2013; Yuan et al., 2013; Schmidt et al., 2014; Chen et al., 2015; García-Perdomo et al., 2017; Tan et al., 2017; Tian et al., 2017; Allen et al., 2019; D’Andrea et al., 2019; Lai et al., 2019; Li et al., 2019; Liao et al., 2019; Liu et al., 2019; Munk et al., 2019; Zhou et al., 2019; Adamou et al., 2020; Madeira et al., 2020; Wang et al., 2020; Zhu et al., 2020; Mykoniatis et al., 2021). Of them, 20 were meta-analyses or network meta-analyses that included a total of 258 relevant meta-analytic effects (Shabsigh et al., 2007; Vecchio et al., 2010; Xiao et al., 2012; Taylor et al., 2013; Yuan et al., 2013; Schmidt et al., 2014; Chen et al., 2015; García-Perdomo et al., 2017; Tan et al., 2017; Tian et al., 2017; D’Andrea et al., 2019; Lai et al., 2019; Li et al., 2019; Liao et al., 2019; Liu et al., 2019; Zhou et al., 2019; Adamou et al., 2020; Madeira et al., 2020; Wang et al., 2020; Zhu et al., 2020; Mykoniatis et al., 2021). The step-by-step screening procedure is illustrated in Supplementary Data S2 and Supplementary Data S3.

**FIGURE 1:**
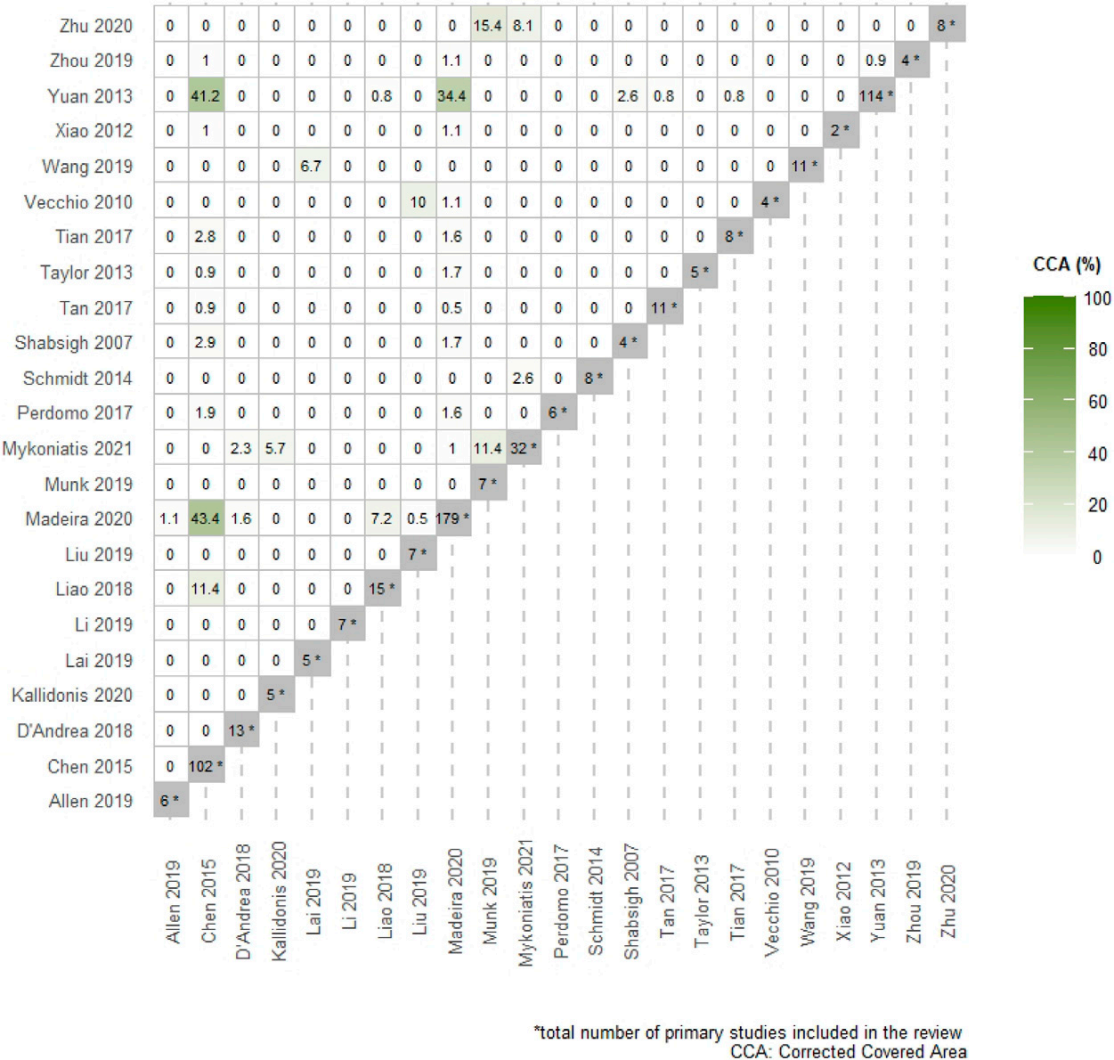
Pairwise intersection heatmap showing the degree of overlap among included systematic reviews. The color-coded cells within the triangular matrix demonstrate the % corrected covered area (CCA) for pairs of SRs. The darker the color, the higher the % CCA. The diagonal grey-colored cells indicate the total number of primary studies included in each review.

The baseline characteristics of the included systematic reviews are presented in Table 1. Overall, a total of 12 studies were funded. Accordingly, the mean overall AMSTAR 2 score of 11 ± 2.9 indicated that the methodological quality of most of the included systematic reviews was generally of sufficient standard (Supplementary Data S4). Across the included studies, the Egger’s test was only reported for 4 meta-analytic effects and no publication bias was detected. The non-reporting of publication bias was predominantly attributed to the low number of studies (<10) included for each meta-analytic effect. Overall, heterogeneity was available for 74 meta-analytic effects, of which 33 displayed substantial heterogeneity I^2^ (>50%). The mean heterogeneity was 36% (range: 0–99%). Furthermore, the overall CCA was 3%, while the CCA for the primary outcome was 37.4%. The corresponding graphical analyses can be seen in Figure 1 and in Supplementary Data S5.

**TABLE 1:** Baseline characteristics of the included systematic reviews. AMSTAR: a measurement tool to assess systematic reviews; CBM: China biological/medicine. CPAP: continuous positive airway pressure; ED: erectile dysfunction; MA: meta-analysis; NA: not available; NMA: network meta-analysis; NOS: Newcastle Ottawa scale; PDE5: phosphodiesterase type 5; RCT: randomized controlled trial; ROB: risk of bias; SR: systematic review.

Study	Design	RCTs (*n*)	Outcomes	Participants (*n*)	Databases searched	Quality assessment of primary studies	Funded	AMSTAR 2 total score
Allen et al. (2019)	SR	6	PDE5 inhibitors vs. placebo in patients with antipsychotic-related sexual dysfunction	175	PubMed, embase, PsycINFO	ROB	No	7
Chen et al. (2015)	NMA	102	PDE5 inhibitors vs. PDE5 inhibitors or placebo	47,626	PubMed	NA	Yes	5
D’Andrea et al. (2019)	MA	13	PDE5 inhibitors vs. placebo for endothelial dysfunction	932	PubMed, Scopus, CINAHL, Science Direct, The Cochrane Library	ROB	No	14
Adamou et al. (2020)	MA	5	PDE5 inhibitors plus a-blocker vs. monotherapy	503	PubMed, Scopus, The Cochrane Library, Web of Science	ROB	No	12.5
Lai et al. (2019)	MA	5	PDE5 inhibitors plus acupuncture vs. PDE5 inhibitors	1751	PubMed, The Cochrane Library, Sinome database, China National Knowledge Infrastructure, Wanfang database, China Science Technology Journal database	ROB	Yes	9.5
Li et al. (2019)	MA	7	PDE5 inhibitors vs. CPAP in patients with obstructive sleep apnea	322	PubMed, embase, Web of Science	ROB	Yes	14
Liao et al. (2019)	NMA	15	PDE5 inhibitors vs. other PDE5 inhibitors or placebo in patients with diabetes	5,274	PubMed, The Cochrane Library, embase, Scopus	ROB	Yes	12.5
Liu et al. (2019)	MA	7	Sildenafil vs. placebo after renal transplantation	332	PubMed, Springer, embase, OVID, The Cochrane Library	ROB	No	13
Madeira et al. (2020)	NMA	179	PDE5 inhibitors vs. PDE5 inhibitors or placebo	50,620	PubMed, Scopus, Web of Science, ClinicalTrials.gov	ROB	Yes	14
Munk et al. (2019)	SR	7	PDE5 inhibitors combination therapy vs. PDE5 inhibitors monotherapy	718	PubMed, embase, Clinical Trials	ROB	No	8
Mykoniatis et al. (2021)	MA	32	PDE5 inhibitors plus another agent vs. PDE5 inhibitors monotherapy	2,788	PubMed, The Cochrane Library, embase	ROB	Yes	15
García-Perdomo et al. (2017)	MA	6	PDE5 inhibitors vs. placebo in patients with spinal cord injury	963	PubMed, The Cochrane Library, embase	ROB	No	11
Schmidt et al. (2014)	MA	8	PDE5 inhibitors plus psychological interventions vs. PDE5 inhibitors monotherapy in patients with psychogenic ED	562	PubMed, embase, PsycINFO, The Cochrane Library, PSYNDEX	ROB	No	12
Shabsigh et al. (2007)	MA	4	Vardenafil in hypertensive patients	2,427	NA	NA	Yes	6.5
Tan et al. (2017)	MA	11	Semen parameters before and after PDE5 inhibitors intake	1,317	PubMed, embase, The Cochrane Library, Web of Science, ClinicalTrials.gov	ROB	Yes	8.5
Taylor et al. (2013)	MA	5	PDE5 inhibitors vs. placebo or other treatments in patients with antidepressant-induced sexual dysfunction	1886	PubMed, The Cochrane Library, embase, CCDANCTR, CINAHL, PsycINFO, ClinicalTrials.gov and ICTRP	ROB	Yes	14.5
Tian et al. (2017)	MA	8	PDE5 inhibitors vs. placebo after nerve-sparing radical prostatectomy	1806	PubMed and embase	ROB	No	10
Vecchio et al. (2010)	MA	4	PDE5 inhibitors vs. placebo in patients with chronic kidney disease	328	PubMed, The Cochrane Library, embase, Cochrane Renal Group’s Specialized Register	ROB	No	7.5
Wang et al. (2020)	MA	11	Tadalafil plus chinese herbal medicine vs. tadalafil monotherapy	903	PubMed, The Cochrane Library, embase, CNKI, Wanfang, Weip database, CBM	ROB	No	12.5
Xiao et al. (2012)	MA	2	Sildenafil vs. placebo or no treatment in patients with multiple sclerosis	420	PubMed The Cochrane Library, embase, CBM	ROB	No	11
Yuan et al. (2013)	NMA	114	PDE5 inhibitors vs. PDE5 inhibitors or placebo	31,195	PubMed, The Cochrane Library, embase	ROB	Yes	13
Zhou et al. (2019)	MA	4	Tadalafil daily vs. tadalafil on demand	1,035	PubMed, The Cochrane Library, embase	ROB	Yes	13
Zhu et al. (2020)	MA	8	PDE5 inhibitors plus testosterone vs. PDE5 inhibitors monotherapy in men with hypogonadism	913	PubMed, The Cochrane Library, embase	ROB	Yes	10

### Efficacy of Phosphodiesterase Type 5 Inhibitors

Three network meta-analyses have addressed the efficacy of all approved PDE5 inhibitors on ED compared to each other or to placebo in the general population. Yuan et al. (2013) explored, for the first time, the efficacy of all approved PDE5 inhibitors without accounting for the different dosages and formulations of each PDE5 inhibitors. Subsequently, Chen et al. (2015) performed a trade-off network meta-analysis to account for these differences and to combine outcomes deriving from the four most important ED questionnaires, namely the IIEF-5 or IIEF–Erectile Function Domain, the Sexual Encounter Profile (SEP) question 2 (SEP-2) and 3 (SEP-3), as well as the Global Assessment Questionnaire question 1 (GAQ-1). However, since most included studies did not provide data on one or more of the four questionnaires, the authors applied multiple imputations to perform these analyses (Chen et al., 2015). In an attempt to harmonize clinical outcomes and heterogeneity, Madeira et al. (2020) recently performed a network meta-analysis including studies that assessed ED only with the IIEF-5 or IIEF–Erectile Function Domain.

**FIGURE 2:**
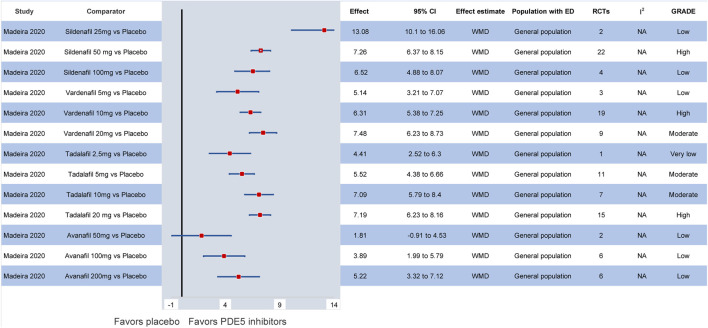
Efficacy of different PDE5 inhibitors in the general population compared to placebo. CI: confidence interval; ED: erectile dysfunction; GRADE: grading of recommendations assessment, development and evaluation; PDE5: phosphodiesterase type 5; RCT: randomized controlled trial; WMD: weighted mean difference.

Combining the outcomes of all available dosages, Yuan et al. (2013) suggested that tadalafil may be considered the most effective compound in the general population, followed by vardenafil. Hence, based on the network meta-analysis by Chen et al. (2015) sildenafil 50 mg (SMD: 0.47, 95% CI: 0.34–0.59) followed by sildenafil 100 mg (SMD: 0.46, 95% CI: 0.35–0.56) were considered the treatments of choice when compared to placebo. In the most recent network meta-analysis by Madeira et al. (2020) sildenafil 25 mg (WMD: 13.08, 95% CI: 10.1–16.06) seemed to be statistically superior to all interventions in improving the IIEF compared to placebo, but studies with low-dose sildenafil are lacking. Still, comparing among different PDE5 inhibitors, sildenafil 50 mg or sildenafil 100 mg seem to be the most effective compounds in the general population. The latter derived, however, predominantly from indirect comparisons among different PDE5 inhibitors (Yuan et al., 2013; Madeira et al., 2020). Of interest, low-dose daily tadalafil may be more effective for the management of ED than high-dose on-demand tadalafil (WMD: 1.24, 95% CI: 0.03–2.44) (Zhou et al., 2019). Overall, in the available studies, the strength of evidence for most pairwise comparisons between different dosages and formulations of PDE5 inhibitors was considered low or very low due to the lack of high-quality RCTs comparing different types of PDE5 inhibitors. All relevant meta-analytic effects are summarized in Figure 2 and Supplementary Data S6.

**FIGURE 3:**
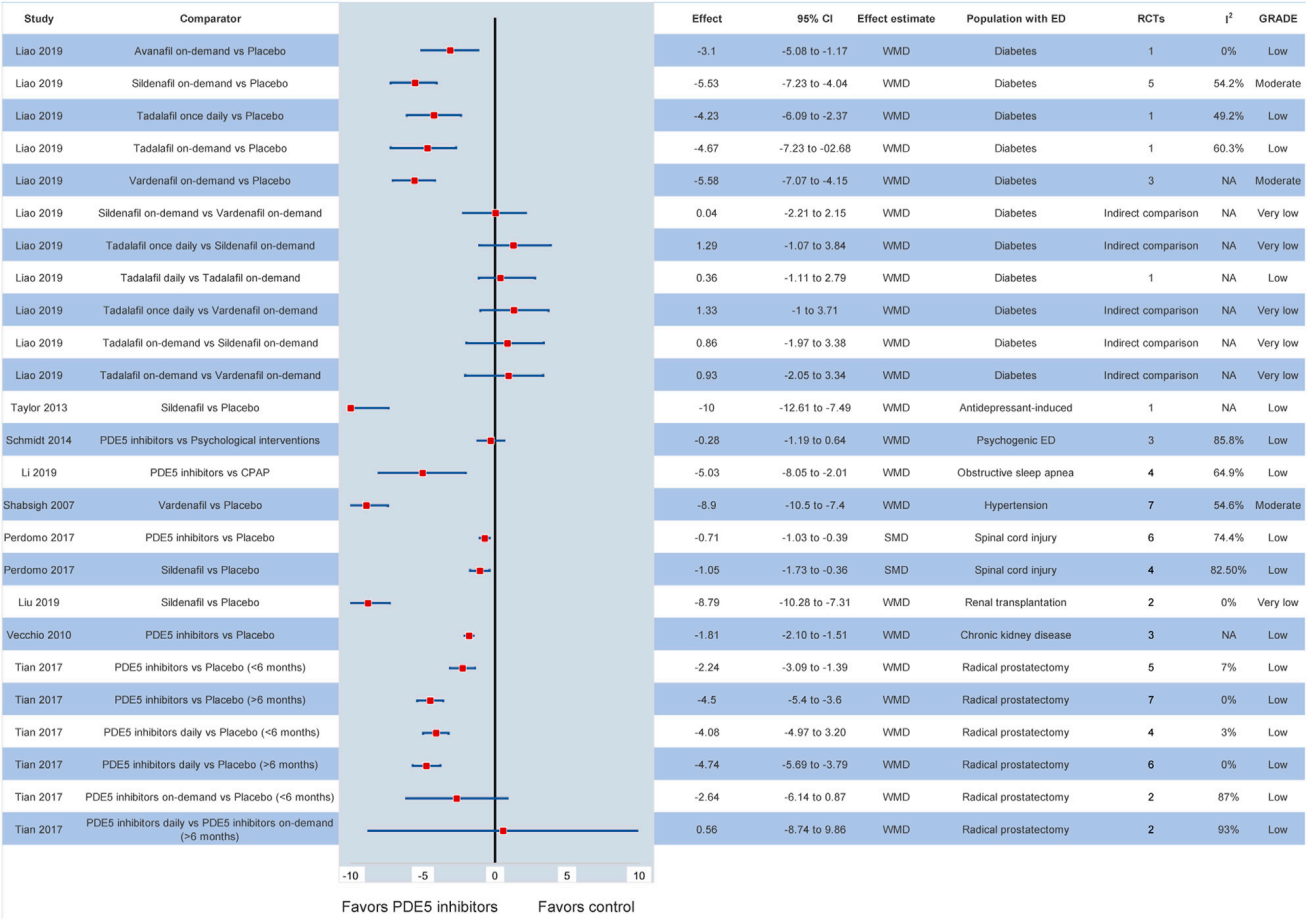
Efficacy of different PDE5 inhibitors in various patient groups. CI: confidence interval; ED: erectile dysfunction; GRADE: grading of recommendations assessment, development and evaluation; NA: not available; PDE5: phosphodiesterase type 5; RCT: randomized controlled trial; SMD: standardized mean difference; WMD: weighted mean difference.

In specific patient populations, the efficacy of PDE5 inhibitors is also well documented. In particular, the available evidence suggests that, in patients with diabetes, all PDE5 inhibitors are superior to placebo (Liao et al., 2019). Comparing among different formulations of PDE5 inhibitors, vardenafil and sildenafil on-demand display the highest efficacy (Liao et al., 2019). In patients with prostatectomy-induced ED, PDE5 inhibitors at any dosage and formulation are superior to placebo and are also recommended as part of penile rehabilitation strategies (Tian et al., 2017). Similarly, their superiority is observed in individuals with hypertension, chronic kidney disease, renal transplantation, spinal cord injury, multiple sclerosis or other neurogenic disorders, obstructive sleep apnea and antidepressant- or antipsychotic-related ED (Shabsigh et al., 2007; Vecchio et al., 2010; Xiao et al., 2012; Taylor et al., 2013; García-Perdomo et al., 2017; Allen et al., 2019; Li et al., 2019; Liu et al., 2019). Of note, in patients with psychogenic ED, the current evidence indicates that no significant differences are demonstrated between PDE5 inhibitor monotherapy and psychological interventions (WMD: −0.28, 95% CI: −1.19–0.64) (Schmidt et al., 2014). Nevertheless, the level of evidence for most outcomes based on the GRADE approach was deemed low or very low. The corresponding meta-analytic effects are presented in Figure 3.

### Safety of Phosphodiesterase Type 5 Inhibitors

Compared to placebo, all dosages and formulations of PDE5 inhibitors present more treatment-related adverse events based on the three network meta-analyses by Yuan et al. (2013), Chen et al. (2015), and Madeira et al. (2020). Hence, the ORs for developing severe adverse events, compared to placebo, do not reach statistical significance for all dosages of PDE5 inhibitors. The within group safety analysis demonstrated that there are no major differences among different PDE5 inhibitors in the general population. Still, sildenafil 100 mg seems to be associated with more treatment-related adverse events and discontinuations due to adverse events (Madeira et al., 2020). Of note, PDE5 inhibitors do not seem to significantly improve endothelial function compared to placebo both in the general population and in patients with diabetes (D’Andrea et al., 2019). Similarly, PDE5 inhibitors do not affect semen parameters (Tan et al., 2017). The relevant comparisons are available in Supplementary Data S7.

### Role of Phosphodiesterase Type 5 Inhibitors as Part of Combination Treatment

**FIGURE 4:**
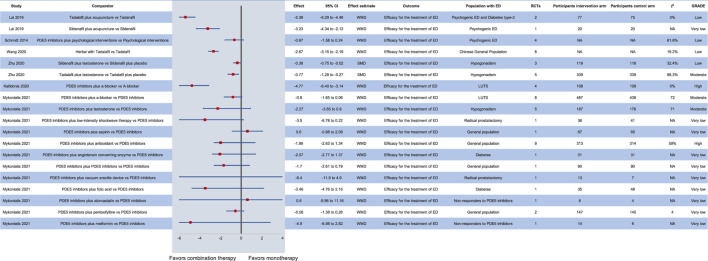
Efficacy of combination therapy with PDE5 inhibitors in various patient groups compared to monotherapy. CI: confidence interval; ED: erectile dysfunction; GRADE: grading of recommendations assessment, development and evaluation; LUTS: lower urinary tract symptoms; NA: not available; PDE5: phosphodiesterase type 5; RCT: randomized controlled trial; SMD: standardized mean difference; WMD: weighted mean difference.

The addition of testosterone in patients with ED and hypogonadism treated with PDE5 inhibitors seems to further improve erectile function (Zhu et al., 2020). In particular, among patients receiving transdermal or oral testosterone due to hypogonadism, sildenafil on-demand at a dose of 50 or 100 mg, as well as 5 mg daily tadalafil display a safe and effective profile. Still, no clear recommendations about the optimal type and dose of treatment can be made. On the other hand, although PDE5 inhibitors improve erectile function in patients with lower urinary tract symptoms (WMD: −4.77, 95% CI: −6.40–−3.14), the addition of a-blockers does not further ameliorate symptoms (WMD: −0.8, 95% CI: −1.65–0.06) (Adamou et al., 2020). In the Chinese general population, the combination of Chinese herbal medicine and tadalafil compared to tadalafil monotherapy may provide beneficial outcomes on ED (WMD: −2.67, 95% CI: −3.15 to −2.19) (Wang et al., 2020). In patients with psychogenic ED, the addition of acupuncture to tadalafil or sildenafil seems to further improve erectile function (Lai et al., 2019), while the addition of PDE5 inhibitors to psychological interventions does not improve symptoms compared to psychological interventions alone (Schmidt et al., 2014). Interestingly, adding to a PDE5 inhibitor: a second PDE5 inhibitor, low-intensity shockwave therapy, a vacuum erectile device or antioxidants is effective in patients with ED, while folic acid, metformin and angiotensin-converting enzyme inhibitors may be effective in some patients with ED but the evidence is still scarce (Munk et al., 2019). The effect of all available combination therapies with PDE5 inhibitors is presented in Figure 4.

## Discussion

Our overview of systematic reviews summarizes the available evidence about PDE5 inhibitors and demonstrates their safety and efficacy both as monotherapy and as part of combination therapy. In patients prioritizing high efficacy with tolerability, sildenafil at low doses (25 or 50 mg) followed by tadalafil may be considered the first-line ED treatment. Still, this observation derives predominantly from indirect comparisons with placebo, since RCTs directly comparing the available PDE5 inhibitors are scarce. PDE5 inhibitors are also effective and safe in almost all patient groups with organic ED, including difficult-to-treat subgroups, such as individuals with diabetes, hypertension, chronic kidney disease, prostatectomy-induced ED and neurological disorders. Still, PDE5 inhibitors do not seem to improve endothelial function.

The development and launch of PDE5 inhibitors more than 2 decades ago have brought upon a revolution for the management of ED (Goldstein et al., 1998). Their safety profile, rapid efficacy, convenient oral administration, availability on an over-the-counter basis and relatively low cost has made them one of the most commonly administered medications (Martin et al., 2013). It should be stressed that low-dose PDE5 inhibitors such as sildenafil 25 mg or tadalafil 5 mg seem to combine high efficacy with lower treatment-related adverse events compared to the same compounds at higher dose. Thus, further RCTs comparing the safety and efficacy profile of PDE5 inhibitors at low dose versus PDE5 inhibitors at high dose are necessary.

PDE5 inhibitors induce corporeal smooth muscle relaxation that increases arterial blood flow, which is followed by compression of the sub-tunical venous plexus and, in turn, leads to erection of the penis (Lue, 2000). Animal models have demonstrated that chronic daily PDE5 inhibitor intake may improve cavernosal endothelial function (Behr-Roussel et al., 2005; Kovanecz et al., 2008). However, these findings have not been replicated in humans (Pattanaik et al., 2019). Based on the previous notion, the included meta-analysis about the effect of PDE5 inhibitors on endothelial function demonstrated that PDE5 inhibitors do not seem to significantly ameliorate endothelial dysfunction and highlighted the need for high-quality primary studies (D’Andrea et al., 2019). In this context, daily tadalafil may be equally effective as on-demand tadalafil (Porst et al., 2014) and the combination of daily tadalafil with on-demand sildenafil may further improve erectile function, especially in patients with severe ED (Cui et al., 2015).

Given that combination treatment with PDE5 inhibitors and testosterone is safe and effective compared to monotherapy of PDE5 inhibitors (Spitzer et al., 2012), it may be advised to initially prefer combination therapy with testosterone and PDE5 inhibitors in patients with hypogonadism (Tsertsvadze et al., 2009). Still, studies exploring the optimal dose and form of administration of both PDE5 inhibitors and testosterone are mandatory. On the contrary, the addition of a-blockers to PDE5 inhibitors does not seem to improve symptoms compared to monotherapy of PDE5 inhibitors in patients with lower urinary tract symptoms and ED. Nevertheless, due to the paucity of available evidence, future relevant studies are expected with great interest.

In non-responders to PDE5 inhibitors or in patients with difficult-to-treat ED, the addition of low-intensity shockwave therapy, a vacuum erectile device or antioxidants may further improve erectile function without increasing the number of adverse events (Morano et al., 2007; Engel, 2011; Baccaglini et al., 2020). However, it should be noted that no exact definitions of non-responders to PDE5 inhibitors and of patients with difficult-to-treat ED exist. Non-responders to PDE5 inhibitors are considered all patients on regular PDE5 inhibitors that abandon treatment due to inefficacy. In such cases, the partner and/or the circumstances surrounding the sexual encounter should be also taken into consideration before classifying somebody as non-responder (Hatzichristou et al., 2005). In particular, one disadvantage of sildenafil is that it acts approximately 1 h after intake and demands avoidance of food or alcohol, which alters the sexual encounter into a timed activity (McCullough et al., 2002). Accordingly, difficult-to-treat ED is defined as the ED that is unresponsive, refractory or relapsing to PDE5 inhibitors. Of note, in this setting, the exclusion of psychogenic ED is necessary, given that PDE5 inhibitors are ineffective (Burnett et al., 2018). In cases of documented difficult-to-treat ED, the increase of the initial PDE5 inhibitor dose may provide some short-term efficacy, but soon clinicians should opt for combination therapies (Salonia et al., 2021). Nevertheless, it should be highlighted that studies evaluating the optimal combination strategies are scarce.

Patients treated with PDE5 inhibitors display high discontinuation rates due to adverse events or inefficacy and, thus, research on novel ED treatment is imperative (Burnett and Hellstrom, 2012). Promising single or combination treatment modalities that may comprise growth factor therapy, stem cell therapy, or even gene therapy and tissue engineering may make their way through the clinical pipeline (Poulios et al., 2021; Raheem et al., 2021). Consequently, studies comparing the efficacy and safety of such treatment strategies in the form of monotherapy or combination therapy with PDE5 inhibitors are mandatory.

### Limitations

The findings of the present study should be interpreted with respect to some limitations. Due to the plethora of primary studies and due to the complexity of some meta-analytic effects, we did not review the primary studies included in each systematic review and did not consider further primary studies published after these systematic reviews. Therefore, for all measures and outcomes, we relied on the information presented in each included systematic review. Even though we included only systematic reviews and meta-analyses of RCTs to provide high level of evidence, the impact of observational, real-world data on the safety and efficacy of PDE5 inhibitors remains uncaptured. Importantly, some systematic reviews and meta-analyses were of low methodological quality, while others displayed high quality and significantly contributed to our results. Based on the previous notion, it should be stressed that, for each outcome, the most methodologically rigorous study based on study design, protocol existence, duplication of data extraction, application of recommended tools and performance of sophisticated statistical analyses was only included. Still, despite our objective criteria, some important studies might have been excluded. Moreover, it was beyond the scope of this review to explore the role of PDE5 inhibitors in disorders not relevant to ED such as premature ejaculation, priapism or pulmonary hypertension. Finally, it should be stressed that all overviews of systematic reviews suffer from the limitation that they summarize a broad topic and much of the information provided in individual studies or systematic reviews cannot be reported in detail.

## Conclusion

This overview of systematic reviews suggests that, although the efficacy and safety of PDE5 inhibitors, compared to placebo, is well-documented both in the general population and in most patient groups with difficult-to-treat ED, the vast amount of existing evidence comparing different PDE5 inhibitors is low. Therefore, high-quality, head-to-head, multicenter RCTs comparing different PDE5 inhibitors are necessary to determine their ideal dosage and formulation based on their safety and efficacy profile. Nevertheless, according to our findings, sildenafil or tadalafil at low dose seem to display high efficacy and safety. Moreover, combination therapy with PDE5 inhibitors plus other effective agents has emerged as a promising treatment modality in patients with refractory, complex or difficult-to-treat ED. Still, further studies producing evidence on the optimal treatment formulation are warranted to establish combination therapy as first-line treatment for ED.

## Data Availability

The original contributions presented in the study are included in the article/Supplementary Material, further inquiries can be directed to the corresponding author.
