# Supplementary material for: The Effect of Phosphodiesterase-type 5 Inhibitors on Erectile Function: An Overview of Systematic Reviews
Source: Front Pharmacol. 2021 Sep 7;12:735708. doi: 10.3389/fphar.2021.735708 (PMC8452927; doi:10.3389/fphar.2021.735708)
Supplement: Supplementary file 1 [file DataSheet1.docx]

Data Supplement

Table of Contents

[Data Supplement 1: PubMed search syntax and search string 2](#_Toc75100525)

[Data Supplement 3: References of all excluded studies with reasons for exclusion 5](#_Toc75100526)

[Data Supplement 4: Quality assessment of the included systematic reviews 10](#_Toc75100527)

[Data Supplement 5: Graphical presentation of corrected covered area for the primary outcome 11](#_Toc75100528)

[Data Supplement 6: Efficacy of different PDE5 inhibitors compared to each other 13](#_Toc75100529)

[Data Supplement 7: Safety of different PDE5 inhibitors 14](#_Toc75100530)

# Data Supplement 1: PubMed search syntax and search string

**Search syntax**

**ID Search**

#1 Sildenafil [All Fields]

#2 Avanafil [All Fields]

#3 Tadalafil [All Fields]

#4 Vardenafil [All Fields]

#5 Mirodenafil [All Fields]

#6 Lodenafil [All Fields]

#7 Udenafil [All Fields]

#8 Phosphodiesterase-5 [All Fields]

#9 Phosphodiesterase 5 [All Fields]

#10 Phosphodiesterase Five [All Fields]

#11 Sildenafil Citrate [MeSH Terms]

#12 Phosphodiesterase 5 Inhibitors [MeSH Terms]

#13 OR #1-12

#14 Sexual [All Fields]

#15 Orgasm [All Fields]

#16 Erectile [All Fields]

#17 Erection [All Fields]

#18 Impotence [All Fields]

#19 IIEF [All Fields]

#20 Orgasm [MeSH Terms]

#21 Erectile Dysfunction [MeSH Terms]

#22 Penile Erection [MeSH Terms]

#23 OR #14-22

#24 Meta-Analysis [All Fields]

#25 Metanalysis [All Fields]

#26 Meta Analysis [All Fields]

#27 Meta-analysis [Publication Type]

#28 Systematic Review [All Fields]

#29 Systematic Review [Publication Type]

#30 OR #24-29

#31 #13 AND #23 AND #30

**Search string**

("sildenafil citrate"[MeSH Terms] OR ("sildenafil"[All Fields] AND "citrate"[All Fields]) OR "sildenafil citrate"[All Fields] OR "sildenafil"[All Fields] OR "sildenafil s"[All Fields] OR ("avanafil"[Supplementary Concept] OR "avanafil"[All Fields]) OR ("vardenafil dihydrochloride"[MeSH Terms] OR ("vardenafil"[All Fields] AND "dihydrochloride"[All Fields]) OR "vardenafil dihydrochloride"[All Fields] OR "vardenafil"[All Fields]) OR ("tadalafil"[MeSH Terms] OR "tadalafil"[All Fields]) OR ("mirodenafil"[Supplementary Concept] OR "mirodenafil"[All Fields]) OR "lodenafil"[All Fields] OR ("udenafil"[Supplementary Concept] OR "udenafil"[All Fields]) OR ("cyclic nucleotide phosphodiesterases, type 5"[MeSH Terms] OR "type 5 cyclic nucleotide phosphodiesterases"[All Fields] OR "phosphodiesterase 5"[All Fields] OR ("cyclic nucleotide phosphodiesterases, type 5"[MeSH Terms] OR "type 5 cyclic nucleotide phosphodiesterases"[All Fields] OR "phosphodiesterase 5"[All Fields]) OR (("phosphoric diester hydrolases"[MeSH Terms] OR ("phosphoric"[All Fields] AND "diester"[All Fields] AND "hydrolases"[All Fields]) OR "phosphoric diester hydrolases"[All Fields] OR "phosphodiesterase"[All Fields] OR "phosphodiesterases"[All Fields]) AND "five"[All Fields]) OR "phosphodiesterase 5 inhibitors"[MeSH Terms])) AND ("sexual behavior"[MeSH Terms] OR ("sexual"[All Fields] AND "behavior"[All Fields]) OR "sexual behavior"[All Fields] OR "sexual"[All Fields] OR "sexually"[All Fields] OR "sexualities"[All Fields] OR "sexuality"[MeSH Terms] OR "sexuality"[All Fields] OR "sexualization"[All Fields] OR "sexualize"[All Fields] OR "sexualized"[All Fields] OR "sexualizing"[All Fields] OR "sexuals"[All Fields] OR ("orgasm"[MeSH Terms] OR "orgasm"[All Fields] OR "orgasmic"[All Fields] OR "orgasms"[All Fields]) OR ("erectility"[All Fields] OR "penile erection"[MeSH Terms] OR ("penile"[All Fields] AND "erection"[All Fields]) OR "penile erection"[All Fields] OR "erectile"[All Fields]) OR ("erections"[All Fields] OR "erective"[All Fields] OR "penile erection"[MeSH Terms] OR ("penile"[All Fields] AND "erection"[All Fields]) OR "penile erection"[All Fields] OR "erection"[All Fields]) OR ("erectile dysfunction"[MeSH Terms] OR ("erectile"[All Fields] AND "dysfunction"[All Fields]) OR "erectile dysfunction"[All Fields] OR "impotence"[All Fields] OR "impotent"[All Fields] OR "impotency"[All Fields]) OR "IIEF"[All Fields] OR "orgasm"[MeSH Terms] OR "erectile dysfunction"[MeSH Terms] OR "penile erection"[MeSH Terms]) AND ("systematic review"[Publication Type] OR "systematic reviews as topic"[MeSH Terms] OR "systematic review"[All Fields] OR ("meta analysis"[Publication Type] OR "meta analysis as topic"[MeSH Terms] OR "meta analysis"[All Fields]))

The search strategy was modified accordingly for the other databasesData Supplement 2: Flow diagram of study selection process

Data Supplement 2: Flow diagram of study selection process. MA: meta-analysis; RCT: randomized controlled trial; SR: systematic review.

# Data Supplement 3: References of all excluded studies with reasons for exclusion

Systematic reviews with no relevant outcome

[1] Barnes H, Brown Z, Burns A, Williams T. Phosphodiesterase 5 inhibitors for pulmonary hypertension. Cochrane Database Syst Rev 2019. https://doi.org/10.1002/14651858.CD012621.pub2.

[2] Martin AL, Huelin R, Wilson D, Foster TS, Mould JF. A Systematic Review Assessing the Economic Impact of Sildenafil Citrate (Viagra®) in the Treatment of Erectile Dysfunction. J Sex Med 2013;10:1389–400. https://doi.org/10.1111/jsm.12068.

[3] Morris DE, Emami B, Mauch PM, Konski AA, Tao ML, Ng AK, et al. Evidence-based review of three-dimensional conformal radiotherapy for localized prostate cancer: An ASTRO outcomes initiative. Int J Radiat Oncol Biol Phys 2005;62:3–19. https://doi.org/10.1016/j.ijrobp.2004.07.666.

[4] Stratton H, Sansom J, Brown-Major A, Anderson P, Ng L. Interventions for sexual dysfunction following stroke. Cochrane Database Syst Rev 2020. https://doi.org/10.1002/14651858.CD011189.pub2.

[5] Vreugdenhil S, Weidenaar AC, de Jong IJ, van Driel MF. Sleep-Related Painful Erections: A Meta-Analysis on the Pathophysiology and Risks and Benefits of Medical Treatments. J Sex Med 2018;15:5–19. https://doi.org/10.1016/j.jsxm.2017.11.006.

Overlapping systematic reviews

[1] Alhathal N, Elshal AM, Carrier Dr. S, Carrier S. Synergetic effect of testosterone and phophodiesterase-5 inhibitors in hypogonadal men with erectile dysfunction: A systematic review. Can Urol Assoc J = J l’Association Des Urol Du Canada 2012;6:269–74. https://doi.org/10.5489/cuaj.11291.

[2] Berner MM, Hagen M, Kriston L. Management of sexual dysfunction due to antipsychotic drug therapy. Cochrane Database Syst Rev 2007. https://doi.org/10.1002/14651858.CD003546.pub2.

[3] Blanker MH, Thomas S, Bohnen AM. Systematic review of Viagra RCTs. Br J Gen Pract 2002;52:329.

[4] Burls A, Gold L, Clark W. Systematic review of randomised controlled trials of sildenafil (Viagra®) in the treatment of male erectile dysfunction. Br J Gen Pract 2001;51:1004–12.

[5] Campos-Juanatey F, Fernandez-Barriales M, Gonzalez M, Portillo-Martin J. Effects of obstructive sleep apnea and its treatment over the erectile function: A systematic review. Asian J Androl 2017;19:303–10. https://doi.org/10.4103/1008-682X.170440.

[6] El-Assmy A. Erectile dysfunction in hemodialysis: A systematic review. World J Nephrol 2012;1:160–5. https://doi.org/10.5527/wjn.v1.i6.160.

[7] Lombardi G, Musco S, Kessler TM, Marzi VL, Lanciotti M, Del Popolo G. Management of sexual dysfunction due to central nervous system disorders: A systematic review. BJU Int 2015;115:47–56. https://doi.org/10.1111/bju.13055.

[8] Marchioni M, De Francesco P, Castellucci R, Papalia R, Sarikaya S, Gómez Rivas J, et al. Management of erectile disfunction following robot-assisted radical prostatectomy: a systematic review. Minerva Urol Nefrol 2020. https://doi.org/10.23736/S0393-2249.20.03780-7.

[9] Melnik T, Soares B, Nasello AG. Psychosocial interventions for erectile dysfunction. Cochrane Database Syst Rev 2007. https://doi.org/10.1002/14651858.CD004825.pub2.

[10] Miles CL, Candy B, Jones L, Williams R, Tookman A, King M. Interventions for sexual dysfunction following treatments for cancer. Cochrane Database Syst Rev 2007. https://doi.org/10.1002/14651858.CD005540.pub2.

[11] Moore RA, Derry S, McQuay HJ. Indirect comparison of interventions using published randomised trials: Systematic review of PDE-5 inhibitors for erectile dysfunction. BMC Urol 2005;5. https://doi.org/10.1186/1471-2490-5-18.

[12] Niccolai Costa AM, de Lima MS, de Jesus Mari J, Costa AMN, Lima MS de, Mari J de J, et al. A systematic review on clinical management of antipsychotic-induced sexual dysfunction in schizophrenia. Sao Paulo Med J 2006;124:291–7. https://doi.org/10.1590/S1516-31802006000500012.

[13] Nicolai M, Urkmez A, Sarikaya S, Fode M, Falcone M, Albersen M, et al. Penile Rehabilitation and Treatment Options for Erectile Dysfunction Following Radical Prostatectomy and Radiotherapy: A Systematic Review. Front Surg 2021;8. https://doi.org/10.3389/fsurg.2021.636974.

[14] Park HJ, Moon KH, Lee SW, Lee WK, Kam SC, Lee JH, et al. Mirodenafil for the treatment of erectile dysfunction: a systematic review of the literature. World J Mens Health 2014;32:18–27. https://doi.org/10.5534/wjmh.2014.32.1.18.

[15] Steinke E, Palm Johansen P, Fridlund B, Broström A. Determinants of sexual dysfunction and interventions for patients with obstructive sleep apnoea: A systematic review. Int J Clin Pract 2016;70:5–19. https://doi.org/10.1111/ijcp.12751.

Meta-analyses with no relevant outcome

[1] Adamou C, Ntasiotis P, Athanasopoulos A, Kallidonis P. The hemodynamic interactions of combination therapy with α-blockers and phosphodiesterase-5 inhibitors compared to monotherapy with α-blockers: a systematic review and meta-analysis. Int Urol Nephrol 2020;52:1407–20. https://doi.org/10.1007/s11255-020-02454-6.

[2] Asimakopoulos AD, Miano R, Agrò EF, Vespasiani G, Spera E. Does Current Scientific and Clinical Evidence Support the Use of Phosphodiesterase Type 5 Inhibitors for the Treatment of Premature Ejaculation? A Systematic Review and Meta-analysis. J Sex Med 2012;9:2404–16. https://doi.org/10.1111/j.1743-6109.2011.02628.x.

[3] Castiglione F, Albersen M, Hedlund P, Gratzke C, Salonia A, Giuliano F. Current Pharmacological Management of Premature Ejaculation: A Systematic Review and Meta-analysis. Eur Urol 2016;69:904–16. https://doi.org/10.1016/j.eururo.2015.12.028.

[4] Chang Rhim H, Kim MS, Park YJ, Choi WS, Park HK, Kim HG, et al. The Potential Role of Arginine Supplements on Erectile Dysfunction: A Systemic Review and Meta-Analysis. J Sex Med 2019;16:223–34. https://doi.org/10.1016/j.jsxm.2018.12.002.

[5] Corona G, Rastrelli G, Morgentaler A, Sforza A, Mannucci E, Maggi M. Meta-analysis of Results of Testosterone Therapy on Sexual Function Based on International Index of Erectile Function Scores[Figure presented]. Eur Urol 2017;72:1000–11. https://doi.org/10.1016/j.eururo.2017.03.032.

[6] Jian Z, Wei X, Ye D, Li H, Wang K. Pharmacotherapy of premature ejaculation: a systematic review and network meta-analysis. Int Urol Nephrol 2018;50:1939–48. https://doi.org/10.1007/s11255-018-1984-9.

[7] Kriston L, Harms A, Berner MM. A meta-regression analysis of treatment effect modifiers in trials with flexible-dose oral sildenafil for erectile dysfunction in broad-spectrum populations. Int J Impot Res 2006;18:559–65. https://doi.org/10.1038/sj.ijir.3901479.

[8] Kuang T-G, Wang J, Zhai Z-G, Guo X-H, Wang C. Efficacy and safety of sildenafil therapy in pulmonary artery hypertension: A meta-analysis. Natl Med J China 2007;87:1021–4.

[9] Martyn-St James M, Cooper K, Ren S, Kaltenthaler E, Dickinson K, Cantrell A, et al. Phosphodiesterase Type 5 Inhibitors for Premature Ejaculation: A Systematic Review and Meta-analysis. Eur Urol Focus 2017;3:119–29. https://doi.org/10.1016/j.euf.2016.02.001.

[10] Poolsup N, Suksomboon N, Aung N. Effect of phosphodiesterase-5 inhibitors on glycemic control in person with type 2 diabetes mellitus: A systematic review and meta-analysis. J Clin Transl Endocrinol 2016;6:50–5. https://doi.org/10.1016/j.jcte.2016.11.003.

[11] Sridharan K, Sivaramakrishnan G, Sequeira RP, Al-Khaja KA. Pharmacological interventions for premature ejaculation: a mixed-treatment comparison network meta-analysis of randomized clinical trials. Int J Impot Res 2018;30:215–23. https://doi.org/10.1038/s41443-018-0030-x.

[12] Stridh A, Pontén M, Arver S, Kirsch I, Abé C, Jensen KB, et al. Placebo Responses Among Men With Erectile Dysfunction Enrolled in Phosphodiesterase 5 Inhibitor Trials: A Systematic Review and Meta-analysis. JAMA Netw Open 2020;3:600. https://doi.org/10.1097/JU.0000000000001167.02.

[13] Wu Q, Yang F, Fang Z, Zhao J, Xiaoyan H, Li L. The clinical efficacy and safety of sildenafil in premature ejaculate: A meta-analysis. Int J Clin Exp Med 2017;10:394–401.

[14] Zhang X, Chi N, Sun M, Shan Z, Zhang Y, Cui Y. Phosphodiesterase-5 Inhibitors for Premature Ejaculation: Systematic Review and Meta-Analysis of Placebo-Controlled Trials. Am J Mens Health 2020;14:1557988320916406. https://doi.org/10.1177/1557988320916406.

Overlapping meta-analyses

[1] Balhara YPS, Sarkar S, Gupta R. Phosphodiesterase-5 inhibitors for erectile dysfunction in patients with diabetes mellitus: A systematic review and meta-analysis of randomized controlled trials. Indian J Endocrinol Metab 2015;19:451–61. https://doi.org/10.4103/2230-8210.159023.

[2] Bansal UK, Jones C, Fuller TW, Wessel C, Jackman S V. The Efficacy of Tadalafil Daily vs on Demand in the Treatment of Erectile Dysfunction: A Systematic Review and Meta-analysis. Urology 2018;112:6–11. https://doi.org/10.1016/j.urology.2017.08.031.

[3] Berner MM, Kriston L, Harms A, Rosen RC, Shaw JW, Berner MM, et al. Efficacy of PDE-5-inhibitors for erectile dysfunction. A comparative meta-analysis of fixed-dose regimen randomized controlled trials administering the International Index of Erectile Function in broad-spectrum populations. Int J Impot Res 2006;18:229–35. https://doi.org/10.1038/sj.ijir.3901395.

[4] Chen PC, Wang CC, Tu YK. Combination alpha blocker and phosphodiesterase 5 inhibitor versus alpha-blocker monotherapy for lower urinary tract symptoms associated with benign prostate hyperplasia: A systematic review and meta-analysis. Urol Sci 2020;31:99–107. https://doi.org/10.4103/UROS.UROS_59_19.

[5] Choi H, Kim HJ, Bae JH, Kim JH, Moon DG, Cheon J, et al. A Meta-Analysis of Long- Versus Short-Acting Phosphodiesterase 5 Inhibitors: Comparing Combination Use With α-Blockers and α-Blocker Monotherapy for Lower Urinary Tract Symptoms and Erectile Dysfunction. Int Neurourol J 2015;19:237–45. https://doi.org/10.5213/inj.2015.19.4.237.

[6] Corona G, Isidori AM, Buvat J, Aversa A, Rastrelli G, Hackett G, et al. Testosterone supplementation and sexual function: a meta-analysis study. J Sex Med 2014;11:1577–92. https://doi.org/10.1111/jsm.12536.

[7] Corona G, Rastrelli G, Burri A, Jannini EA, Maggi M. The safety and efficacy of Avanafil, a new 2nd generation PDE5i: Comprehensive review and meta-analysis. Expert Opin Drug Saf 2016;15:237–47. https://doi.org/10.1517/14740338.2016.1130126.

[8] Cui Y-SYS, Li N, Zong H-THT, Yan HLH-L, Zhang Y. Avanafil for male erectile dysfunction: A systematic review and meta-analysis. Asian J Androl 2014;16:472–7. https://doi.org/10.4103/1008-682X.123670.

[9] Cui Y, Liu X, Shi L, Gao Z. Efficacy and safety of phosphodiesterase type 5 (PDE5) inhibitors in treating erectile dysfunction after bilateral nerve-sparing radical prostatectomy. Andrologia 2016;48:20–8. https://doi.org/10.1111/and.12405.

[10] Deng T, Duan X, Liu B, Lan Y, Cai C, Zhang T, et al. Association between phosphodiesterase type 5 inhibitors use and risk of melanoma: a meta-analysis. Neoplasma 2018;65:216–21. https://doi.org/10.4149/neo_2018_170111N23.

[11] Ding H, Du W, Wang H, Zhang L, Wang Z, Du C, et al. Efficacy and safety of udenafil for erectile dysfunction: A meta-analysis of randomized controlled trials. Urology 2012;80:134–9. https://doi.org/10.1016/j.urology.2012.02.014.

[12] Dong L, Zhang X, Yan X, Shen Y, Li Y, Yu X. Effect of phosphodiesterase-5 inhibitors on the treatment of male infertility: A systematic review and meta-analysis. World J Mens Health 2021;39. https://doi.org/10.5534/WJMH.200155.

[13] Dong Y, Hao L, Shi Z, Wang G, Zhang Z, Han C. Efficacy and safety of tadalafil monotherapy for lower urinary tract symptoms secondary to benign prostatic hyperplasia: A meta-analysis. Urol Int 2013;91:10–8. https://doi.org/10.1159/000351405.

[14] Du W, Li J, Fan N, Shang P, Wang Z, Ding H. Efficacy and safety of mirodenafil for patients with erectile dysfunction: A meta-analysis of three multicenter, randomized, double-blind, placebo-controlled clinical trials. Aging Male 2014;17:107–11. https://doi.org/10.3109/13685538.2013.858114.

[15] Fanbin L, Mei Y, Yan Z, Yirong Y, Shaoling Z, Yong C, et al. Efficacy and safety of phosphodiesterase-5 inhibitors for treating erectile dysfunction in kidney transplant recipients: a meta-analysis. Exp Clin Transplant Off J Middle East Soc Organ Transplant 2014;12:184–9. https://doi.org/10.6002/ect.2013.0150.

[16] Feng D, Liu S, Yang Y, Bai Y, Li D, Han P, et al. Generating comprehensive comparative evidence on various interventions for penile rehabilitation in patients with erectile dysfunction after radical prostatectomy: a systematic review and network meta-analysis. Transl Androl Urol 2021;10:109–24. https://doi.org/10.21037/tau-20-892.

[17] Fink HA, Donald R Mac, Rutks IR, Nelson DB, Wilt TJ. Sildenafil for male erectile dysfunction: A systematic review and meta-analysis. Arch Intern Med 2002;162:1349–60. https://doi.org/10.1001/archinte.162.12.1349.

[18] Gacci M, Corona G, Salvi M, Vignozzi L, McVary KT, Kaplan SA, et al. A systematic review and meta-analysis on the use of phosphodiesterase 5 inhibitors alone or in combination with α-blockers for lower urinary tract symptoms due to benign prostatic hyperplasia. Eur Urol 2012;61:994–1003. https://doi.org/10.1016/j.eururo.2012.02.033.

[19] Gong B, Ma M, Xie W, Yang X, Huang Y, Sun T, et al. Direct comparison of tadalafil with sildenafil for the treatment of erectile dysfunction: a systematic review and meta-analysis. Int Urol Nephrol 2017;49:1731–40. https://doi.org/10.1007/s11255-017-1644-5.

[20] Guo B, Chen X, Wang M, Hou H, Zhang Z, Liu M. Comparative effectiveness of tadalafil versus tamsulosin in treating lower urinary tract symptoms suggestive of benign prostate hyperplasia: A meta-analysis of randomized controlled trials. Med Sci Monit 2020;26. https://doi.org/10.12659/MSM.923179.

[21] Jia D-DD, Shuang W-BB, Cheng T, Jia X-MM, Zhang M. Efficacy and safety of phosphodieterase-5 inhibitors for treatment of erectile dysfunction secondary to spinal cord injury: a systemic review and meta-analysis. Spinal Cord 2016;54:494–501. https://doi.org/10.1038/sc.2016.3.

[22] Laydner HK, Oliveira P, Oliveira CRA, Makarawo TP, Andrade WS, Tannus M, et al. Phosphodiesterase 5 inhibitors for lower urinary tract symptoms secondary to benign prostatic hyperplasia: A systematic review. BJU Int 2011;107:1104–9. https://doi.org/10.1111/j.1464-410X.2010.09698.x.

[23] Li J, Peng L, Cao D, He L, Li Y, Wei Q. Avanafil for the Treatment of men With Erectile Dysfunction: A Systematic Review and Meta-analysis of Randomized Controlled Trials. Am J Mens Health 2019;13. https://doi.org/10.1177/1557988319880764.

[24] Li X, Dong Z, Wan Y, Wang Z. Sildenafil versus continuous positive airway pressure for erectile dysfunction in men with obstructive sleep apnea: A meta-analysis. Aging Male 2010;13:82–6. https://doi.org/10.3109/13685530903406789.

[25] Limoncin E, Gravina GL, Corona G, Maggi M, Ciocca G, Lenzi A, et al. Erectile function recovery in men treated with phosphodiesterase type 5 inhibitor administration after bilateral nerve-sparing radical prostatectomy: a systematic review of placebo-controlled randomized trials with trial sequential analysis. Andrology 2017;5:863–72. https://doi.org/10.1111/andr.12403.

[26] Liu L, Zheng S, Han P, Wei Q. Phosphodiesterase-5 inhibitors for lower urinary tract symptoms secondary to benign prostatic hyperplasia: A systematic review and meta-analysis. Urology 2011;77:123–9. https://doi.org/10.1016/j.urology.2010.07.508.

[27] Loeb S, Ventimiglia E, Salonia A, Folkvaljon Y, Stattin P. Meta-Analysis of the Association between Phosphodiesterase Inhibitors (PDE5Is) and Risk of Melanoma. J Natl Cancer Inst 2017;109. https://doi.org/10.1093/jnci/djx086.

[28] Lu X, Han H, Xing N, Tian L. Efficacy of sildenafil citrate in men with erectile dysfunction following bilateral nerve-sparing radical prostatectomy: Systematic review and meta-analysis. Natl Med J China 2015;95:2964–8. https://doi.org/10.3760/cma.j.issn.0376-2491.2015.36.017.

[29] Ma C, Zhang J, Cai Z, Xiong J, Li H. Defining the Efficacy and Safety of Phosphodiesterase Type 5 Inhibitors with Tamsulosin for the Treatment of Lower Urinary Tract Symptoms Secondary to Benign Prostatic Hyperplasia with or without Erectile Dysfunction: A Network Meta-Analysis. Biomed Res Int 2020;2020. https://doi.org/10.1155/2020/1419520.

[30] Ma J, Liu Z, Wu J, Zhou Z, Zhang X, Cui Y, et al. Role of application of tadalafil 5 mg once-daily (≥6 months) in men with erectile dysfunction from six randomized controlled trials. Transl Androl Urol 2020;9:1405–14. https://doi.org/10.21037/tau-19-809.

[31] Markou S, Perimenis P, Gyftopoulos K, Athanasopoulos A, Barbalias G. Vardenafil (Levitra) for erectile dysfunction: A systematic review and meta-analysis of clinical trial reports. Int J Impot Res 2004;16:470–8. https://doi.org/10.1038/sj.ijir.3901258.

[32] Melnik T, Soares BGOO, Nasello AG. The effectiveness of psychological interventions for the treatment of erectile dysfunction: Systematic review and meta-analysis, including comparisons to sildenafil treatment, intracavernosal injection, and vacuum devices. J Sex Med 2008;5:2562–74. https://doi.org/10.1111/j.1743-6109.2008.00872.x.

[33] Montorsi F, McCullough A. Efficacy of sildenafil citrate in men with erectile dysfunction following radical prostatectomy: A systematic review of clinical data. J Sex Med 2005;2:658–67. https://doi.org/10.1111/j.1743-6109.2005.00117.x.

[34] Moore RA, Edwards JE, McQuay HJ. Sildenafil (Viagra) for male erectile dysfunction: A meta-analysis of clinical trial reports. BMC Urol 2002;2:1–12. https://doi.org/10.1186/1471-2490-2-6.

[35] Paduch DA, Bolyakov A, Polzer PK, Watts SD. Effects of 12 weeks of tadalafil treatment on ejaculatory and orgasmic dysfunction and sexual satisfaction in patients with mild to severe erectile dysfunction: Integrated analysis of 17 placebo-controlled studies. BJU Int 2013;111:334–43. https://doi.org/10.1111/j.1464-410X.2012.11656.x.

[36] Penedones A, Alves C, Batel Marques F. Risk of nonarteritic ischaemic optic neuropathy with phosphodiesterase type 5 inhibitors: a systematic review and meta-analysis. Acta Ophthalmol 2020;98:22–31. https://doi.org/10.1111/aos.14253.

[37] Peng Z, Yang L, Dong Q, Wei Q, Liu L, Yang B. Efficacy and Safety of Tadalafil Once-a-Day versus Tadalafil On-Demand in Patients with Erectile Dysfunction: A Systematic Review and Meta-Analyses. Urol Int 2017;99:343–52. https://doi.org/10.1159/000477496.

[38] Qiu S, Tang Z, Deng L, Liu L, Han P, Yang L, et al. Comparisons of regular and on-demand regimen of PED5-Is in the treatment of ED after nerve-sparing radical prostatectomy for Prostate Cancer. Sci Rep 2016;6. https://doi.org/10.1038/srep32853.

[39] Sari Motlagh R, Abufaraj M, Yang L, Mori K, Pradere B, Laukhtina E, et al. Penile Rehabilitation Strategy after Nerve Sparing Radical Prostatectomy: A Systematic Review and Network Meta-Analysis of Randomized Trials. J Urol 2021;205:1018–30. https://doi.org/10.1097/JU.0000000000001584.

[40] Shah PC, Trivedi NA. A meta-analysis on efficacy and tolerability of sildenafil for erectile dysfunction in patients with diabetes mellitus. Indian J Sex Transm Dis AIDS 2018;39:1–6. https://doi.org/10.4103/ijstd.IJSTD_99_17.

[41] Tang H, Wu W, Fu S, Zhai S, Song Y, Han J. Phosphodiesterase type 5 inhibitors and risk of melanoma: A meta-analysis. J Am Acad Dermatol 2017;77:480-488.e9. https://doi.org/10.1016/j.jaad.2017.04.1129.

[42] Tsertsvadze A, Fink HA, Yazdi F, MacDonald R, Bella AJ, Ansari MT, et al. Oral Phosphodiesterase-5 Inhibitors and Hormonal Treatments for Erectile Dysfunction: A Systematic Review and Meta-analysis. Ann Intern Med 2009;151:650. https://doi.org/10.7326/0003-4819-151-9-200911030-00150.

[43] Tsertsvadze A, Yazdi F, Fink HA, MacDonald R, Wilt TJ, Bella AJ, et al. Oral Sildenafil Citrate (Viagra) for Erectile Dysfunction: A Systematic Review and Meta-analysis of Harms. Urology 2009;74:831-836.e8. https://doi.org/10.1016/j.urology.2009.04.026.

[44] Vardi M, Nini A. Phosphodiesterase inhibitors for erectile dysfunction in patients with diabetes mellitus. Cochrane Database Syst Rev 2007. https://doi.org/10.1002/14651858.CD002187.pub3.

[45] Wang H, Guo B, Huang Z, Zhao X, Ji Z. Vardenafil in the Treatment of Male Erectile Dysfunction: A Systematic Review and Meta-Analysis. Adv Ther 2021;38:1301–13. https://doi.org/10.1007/s12325-020-01559-9.

[46] Wang H, Yuan J, Hu X, Tao K, Liu J, Hu D. The effectiveness and safety of avanafil for erectile dysfunction: A systematic review and meta-analysis. Curr Med Res Opin 2014;30:1565–71. https://doi.org/10.1185/03007995.2014.909391.

[47] Wang JJ, Shen Y, Wang JJ, Xue Y, Liao L, Thapa S, et al. Relation of phosphodiesterase type 5 inhibitors and malignant melanoma: A meta-analysis and systematic review. Oncotarget 2017;8:46461–7. https://doi.org/10.18632/oncotarget.17518.

[48] Wang XX, Wang XX, Liu T, He Q, Wang Y, Zhang X. Systematic review and meta-analysis of the use of phosphodiesterase type 5 inhibitors for treatment of erectile dysfunction following bilateral nerve-sparing radical prostatectomy. PLoS One 2014;9. https://doi.org/10.1371/journal.pone.0091327.

[49] Wang XHXX-H, Wang XHXX-H, Shi MJM-J, Li S, Liu T, Zhang XHX-H. Systematic review and meta-analysis on phosphodiesterase 5 inhibitors and α-adrenoceptor antagonists used alone or combined for treatment of LUTS due to BPH. Asian J Androl 2015;17:1022–32. https://doi.org/10.4103/1008-682X.154990.

[50] Wang Y, Bao Y, Liu J, Duan L, Cui Y. Tadalafil 5 mg Once Daily Improves Lower Urinary Tract Symptoms and Erectile Dysfunction: A Systematic Review and Meta-analysis. LUTS Low Urin Tract Symptoms 2018;10:84–92. https://doi.org/10.1111/luts.12144.

[51] Wu Y, Qu X, Wang Y, Xia J, Gu Y, Qian Q, et al. Effect of phosphodiesterase type 5 inhibitors on prostate cancer risk and biochemical recurrence after prostate cancer treatment: A systematic review and meta-analysis. Andrologia 2019;51. https://doi.org/10.1111/and.13198.

[52] Yan H, Zong H, Cui Y, Li N, Zhang Y. The Efficacy of PDE5 Inhibitors Alone or in Combination with Alpha-Blockers for the Treatment of Erectile Dysfunction and Lower Urinary Tract Symptoms Due to Benign Prostatic Hyperplasia: A Systematic Review and Meta-Analysis. J Sex Med 2014;11:1539–45. https://doi.org/10.1111/jsm.12499.

[53] Yang J, Jian ZY, Wang J. Phosphodiesterase type-5 inhibitors for erectile dysfunction following nerve-sparing radical prostatectomy: A network meta-analysis. Medicine (Baltimore) 2021;100:e23778. https://doi.org/10.1097/MD.0000000000023778.

[54] Yang L, Qian S, Liu L, Pu C, Yuan H, Han P, et al. Phosphodiesterase-5 inhibitors could be efficacious in the treatment of erectile dysfunction after radiotherapy for prostate cancer: A systematic review and meta-analysis. Urol Int 2013;90:339–47. https://doi.org/10.1159/000343730.

[55] Yuan J-QJQ, Mao C, Yang ZYZ-Y, Fu X-HXH, Wong SY, Tang J-LJL. A meta-regression evaluating the effectiveness and prognostic factors of oral phosphodiesterase type 5 inhibitors for the treatment of erectile dysfunction. Asian J Androl 2016;18:60–5. https://doi.org/10.4103/1008-682X.154304.

[56] Zhang J, Li X, Yang B, Wu C, Fan Y, Li H. Alpha-blockers with or without phosphodiesterase type 5 inhibitor for treatment of lower urinary tract symptoms secondary to benign prostatic hyperplasia: a systematic review and meta-analysis. World J Urol 2019;37:143–53. https://doi.org/10.1007/s00345-018-2370-z.

[57] Zhou Q, Li L. Meta analysis of randomized controlled trials of sildenafil in treatment of male erectile dysfunction. Chinese J Androl 2006;20:45–7.

[58] Zhou Z, Zheng X, Wu J, Gao Z, Xu Z, Cui Y. Meta-Analysis of Efficacy and Safety of Tadalafil Plus Tamsulosin Compared with Tadalafil Alone in Treating Men with Benign Prostatic Hyperplasia and Erectile Dysfunction. Am J Mens Health 2019;13:1557988319882597. https://doi.org/10.1177/1557988319882597.

Systematic reviews or meta-analyses including observational studies

[1] Corona G, Rastrell G, Burri A, Serra E, Gianfrilli D, Mannucci E, et al. First-generation phosphodiesterase type 5 inhibitors dropout: A comprehensive review and meta-analysis. Andrology 2016;4:1002–9. https://doi.org/10.1111/andr.12255.

[2] Del Popolo G, Cito G, Gemma L, Natali A. Neurogenic Sexual Dysfunction Treatment: A Systematic Review. Eur Urol Focus 2020;6:868–76. https://doi.org/10.1016/j.euf.2019.12.002.

[3] Han X, Han Y, Zheng Y, Sun Q, Ma T, Dai L, et al. Use of phosphodiesterase type 5 inhibitors and risk of melanoma: A meta-analysis of observational studies. Onco Targets Ther 2018;11:711–20. https://doi.org/10.2147/OTT.S142637.

[4] He Q, Liao B-HBH, Xiao K-WKW, Zhou L, Feng S-JSJ, Li H, et al. Is there a relationship between phosphodiesterase type 5 inhibitors and biochemical recurrence after radical prostatectomy: a systematic review and meta-analysis. Int Urol Nephrol 2018;50:2113–21. https://doi.org/10.1007/s11255-018-1982-y.

[5] Liu B, Zhu L, Zhong J, Zeng G, Deng T. The Association Between Phosphodiesterase Type 5 Inhibitor Use and Risk of Non-Arteritic Anterior Ischemic Optic Neuropathy: A Systematic Review and Meta-Analysis. Sex Med 2018;6:185–92. https://doi.org/10.1016/j.esxm.2018.03.001.

[6] Manna S, Gray ML, Kaul VF, Wanna G. Phosphodiesterase-5 (PDE-5) Inhibitors and Ototoxicity: A Systematic Review. Otol Neurotol 2019;40:276–83. https://doi.org/10.1097/MAO.0000000000002148.

[7] Schmid FA, Held U, Eberli D, Pape H-C, Halvachizadeh S. Erectile dysfunction and penile rehabilitation after pelvic fracture: a systematic review and meta-analysis. BMJ Open 2021;11:e045117. https://doi.org/10.1136/bmjopen-2020-045117.

# Data Supplement 4: Quality assessment of the included systematic reviews

| Study | Q1 | Q2 | Q3 | Q4 | Q5 | Q6 | Q7 | Q8 | Q9 | Q10 | Q11 | Q12 | Q13 | Q14 | Q15 | Q16 | Total score |
| --- | --- | --- | --- | --- | --- | --- | --- | --- | --- | --- | --- | --- | --- | --- | --- | --- | --- |
| Allen 2019 | 1 | 0 | 0 | 0.5 | 1 | 1 | 0 | 0.5 | 0 | 1 | 0 | 0 | 1 | 0 | 0 | 1 | 7 |
| Chen 2015 | 1 | 0.5 | 0 | 0 | 0 | 1 | 0 | 0.5 | 0 | 0 | 1 | 0 | 0 | 0 | 0 | 1 | 5 |
| D’Andrea 2019 | 1 | 1 | 1 | 1 | 1 | 1 | 0 | 1 | 1 | 0 | 1 | 1 | 1 | 1 | 1 | 1 | 14 |
| Kallidonis 2020 | 1 | 1 | 1 | 0.5 | 1 | 1 | 0 | 1 | 1 | 0 | 1 | 1 | 1 | 1 | 0 | 1 | 12.5 |
| Lai 2019 | 1 | 0 | 1 | 0.5 | 1 | 1 | 0 | 1 | 1 | 0 | 1 | 0 | 0 | 1 | 0 | 1 | 9.5 |
| Li 2019 | 1 | 1 | 1 | 1 | 1 | 1 | 0 | 1 | 1 | 0 | 1 | 1 | 1 | 1 | 1 | 1 | 14 |
| Liao 2019 | 1 | 1 | 1 | 0.5 | 1 | 1 | 0 | 1 | 1 | 0 | 1 | 1 | 1 | 1 | 1 | 0 | 12.5 |
| Liu 2019 | 1 | 0.5 | 1 | 0.5 | 1 | 1 | 0 | 1 | 1 | 0 | 1 | 1 | 1 | 1 | 1 | 1 | 13 |
| Madeira 2020 | 1 | 1 | 1 | 1 | 1 | 1 | 1 | 1 | 1 | 1 | 1 | 1 | 1 | 0 | 0 | 1 | 14 |
| Munk 2019 | 1 | 0.5 | 1 | 0.5 | 1 | 0 | 1 | 1 | 1 | 0 | 0 | 0 | 0 | 0 | 0 | 1 | 8 |
| Mykoniatis 2021 | 1 | 1 | 1 | 1 | 1 | 1 | 1 | 1 | 1 | 0 | 1 | 1 | 1 | 1 | 1 | 1 | 15 |
| Perdomo 2017 | 1 | 0.5 | 1 | 0.5 | 1 | 1 | 0 | 1 | 1 | 0 | 1 | 1 | 1 | 1 | 0 | 0 | 11 |
| Schmidt 2014 | 1 | 0.5 | 1 | 0.5 | 0 | 1 | 0 | 1 | 1 | 1 | 1 | 1 | 1 | 1 | 0 | 1 | 12 |
| Shabsigh 2007 | 1 | 0.5 | 1 | 0 | 0 | 0 | 0 | 1 | 0 | 0 | 1 | 0 | 0 | 1 | 0 | 1 | 6.5 |
| Tan 2017 | 1 | 0.5 | 1 | 0.5 | 1 | 1 | 0 | 0.5 | 0 | 0 | 1 | 0 | 0 | 1 | 0 | 1 | 8.5 |
| Taylor 2013 | 1 | 0.5 | 1 | 1 | 1 | 1 | 1 | 1 | 1 | 0 | 1 | 1 | 1 | 1 | 1 | 1 | 14.5 |
| Tian 2017 | 1 | 0.5 | 1 | 0.5 | 0 | 1 | 0 | 1 | 1 | 0 | 1 | 0 | 0 | 1 | 1 | 1 | 10 |
| Vecchio 2010 | 1 | 0.5 | 1 | 0.5 | 1 | 1 | 0 | 1 | 0.5 | 0 | 0 | 0 | 0 | 0 | 0 | 1 | 7.5 |
| Wang 2020 | 1 | 0.5 | 1 | 0.5 | 1 | 1 | 0 | 0.5 | 1 | 0 | 1 | 1 | 1 | 1 | 1 | 1 | 12.5 |
| Xiao 2012 | 1 | 1 | 1 | 1 | 1 | 1 | 1 | 1 | 1 | 1 | 1 | 0 | 0 | 0 | 0 | 1 | 12 |
| Yuan 2013 | 1 | 0 | 1 | 1 | 1 | 1 | 0 | 1 | 1 | 0 | 1 | 1 | 1 | 1 | 1 | 1 | 13 |
| Zhou 2019 | 1 | 0.5 | 1 | 0.5 | 1 | 1 | 0 | 1 | 1 | 0 | 1 | 1 | 1 | 1 | 1 | 1 | 13 |
| Zhu 2020 | 1 | 0.5 | 1 | 0.5 | 0 | 0 | 0 | 1 | 1 | 0 | 1 | 1 | 0 | 1 | 1 | 1 | 10 |

Data Supplement 4: Quality assessment of the included systematic reviews based on AMSTAR 2.

# Data Supplement 5: Graphical presentation of corrected covered area for the primary outcome


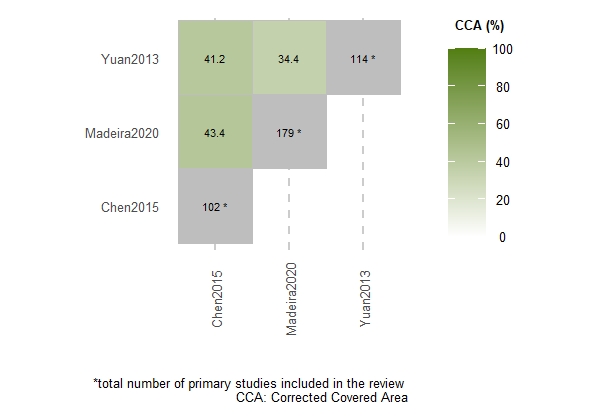


Data Supplement 5: Pairwise intersection heatmap showing the degree of overlap for the primary outcome. The color-coded cells within the triangular matrix demonstrate the % corrected covered area (CCA) for pairs of SRs. The darker the color, the higher the % CCA. The diagonal grey-colored cells indicate the total number of primary studies included in each review.

# Data Supplement 6: Efficacy of different PDE5 inhibitors compared to each other

Data Supplement 6: Efficacy of different PDE5 inhibitors in the general population compared to each other. CI: confidence interval; ED: erectile dysfunction; GRADE: grading of recommendations assessment, development and evaluation; NA: not available; PDE5: phosphodiesterase type 5; RCT: randomized controlled trial; WMD: weighted mean difference.

# Data Supplement 7: Safety of different PDE5 inhibitors

Data Supplement 7.1: Safety of different PDE5 inhibitors in the general population compared to placebo. AE: adverse event; CI: confidence interval; ED: erectile dysfunction; GRADE: grading of recommendations assessment, development and evaluation; NA: not available; OR: odds ratio; PDE5: phosphodiesterase type 5; RCT: randomized controlled trial.

Data Supplement 7.2: Safety of different PDE5 inhibitors in the general population compared to each other. AE: adverse event; CI: confidence interval; ED: erectile dysfunction; GRADE: grading of recommendations assessment, development and evaluation; NA: not available; OR: odds ratio; PDE5: phosphodiesterase type 5; RCT: randomized controlled trial.
